# mmW Rotman Lens-Based Sensing: An Investigation Study

**DOI:** 10.3390/s21041163

**Published:** 2021-02-07

**Authors:** Waleed T. Sethi, Ahmed B. Ibrahim, Khaled Issa, Saleh A. Alshebeili

**Affiliations:** 1 Faculty of Electrical Engineering, Ghulam Ishaq Khan Institute of Engineering Sciences and Technology, Swabi 23640, Pakistan; 2 KACST-TIC in Radio Frequency and Photonics (RFTONICS), King Saud University, Riyadh 11421, Saudi Arabia; ahahmed@ksu.edu.sa; 3 Laboratory of Electronics and Microelectronics, Department of Physics, Faculty of Sciences of Monastir, University of Monastir, Monastir 5000, Tunisia; khaled.issa@yahoo.fr; 4 Electrical Engineering Department, King Saud University, Riyadh 11421, Saudi Arabia; dsaleh@ksu.edu.sa

**Keywords:** 28 GHz, millimeter wave (mmW), Rotman lens, sensor

## Abstract

A Rotman lens is a wideband true-time delay device. Due to its simplistic structure with wave/signal routing capabilities, it has been widely utilized as a beamforming device in numerous communication systems. Since the basic Rotman lens design incorporates multiple input, output, and dummy ports, in this study, and for the first time, we utilized a Rotman lens as a sensor. The main idea was to gather abundant information from available Rotman lens ports to obtain better sensing performance. The realized lens is optimized to work in the millimeter wave (mmW) band from 27 to 29 GHz with a focus on a central frequency of 28 GHz. The design has a footprint of 140 × 103 × 0.8 mm^3^. The polarity correlator was used to characterize the material under investigation.

## 1. Introduction

Microwave/RF sensors have gained much attention in the research community compared to traditional wire-based and magnetic induction sensing due to their usage in minutely detecting and characterizing materials wirelessly [1]. Due to the success rate of these devices in sensing minute changes, the instrument and material characterization industry is working hard to improve the existing tools. The RF sensors are utilized in sensing tiny changes, whether at the molecular level, like sensing glucose solution concentration [2], or at the physical level, by assisting in the daily living healthcare of patients and healthy beings alike [3]. This is to fulfill the requirements for characterizing materials in the field of food processing monitoring, agricultural research, seismic detection, and chemical composition and biosensor applications. Characterizing a material is of crucial importance in understanding the scientific logic behind it and allowing oneself to further investigate the material or sample properties firsthand.

Numerous sensor designs have been realized that efficiently and precisely perform sensing tasks [4,5,6]. Some examples include the technology of split ring resonators, transmission lines, reflection-based resonators, dielectric resonators, substrate integrated waveguide technology, and resonant perturbation methods [7]. Some types of these sensors operate with a single port for analysis and others utilize two-port networks, based on system requirements. Nevertheless, each sensor performs well for specific frequency bands, selective physical properties of materials, and narrow applications defined by its own constraints. Furthermore, this group of microwave methods are dependent on the s-parameter behavior to detect and extract the properties of materials. The amount of change in frequency shifts of s-parameters and the E-field distributions on the sensor structure would reflect the accuracy and sensitivity of the sensor.

A beamforming technique that offers simplicity in design, cost efficiency, ease of implementation, and wide scanning ability is offered by the Rotman lens, proposed in the year 1963 [8]. With its simple geometric design, the Rotman lens can easily steer beams in the desired direction, providing the same results as complex phase shifters [9]. Being a true-time delay device, it also offers a wide band characteristic as it is frequency independent. The design is widely appreciated in mmW band applications due to minimal surface and conduction losses [10,11].

In this study, and for the first time, we designed a basic Rotman lens and investigated its use as a sensor. The main idea was to take advantage of the wideband characteristics of the Rotman lens in order to gather abundant data captured from multiple ports for signal processing analysis. The focus was not on the beam steering capabilities of the Rotman lens but rather on sensing the changes in frequency responses of different materials over the entire spectrum band of interest to determine the parameter characterizing a given material. The proposed Rotman lens sensor has five beam ports, six array ports, and four dummy ports. The sensor design is optimized to work in the mmW band, i.e., from 27 to 29 GHz with focus on the central frequency of 28 GHz. The response of each port of the Rotman lens sensor was considered in terms of its s-parameters. Next, the sensor was loaded with a cylindrical beaker containing aqueous solutions. The beaker was placed on the sensor. The responses of multiple ports were investigated to confirm whether a Rotman lens can be used as an alternate and viable candidate for sensing applications.

The paper is organized as follows. Section 2 describes the basic principle of a Rotman lens and the geometric design of the proposed mmW Rotman lens sensor. It also presents the fabricated prototype and discusses in detail the s-parameter characterization in terms of reflection and coupling coefficients and surface current distributions. Section 3 describes the preparation method of the aqueous solutions and the test of the Rotman lens as a sensor by loading it with various aqueous solutions. For proof of concept, the sensing was characterized between port numbers (P3–P9). Section 4 considers the signal processing aspect of the design in analyzing the abundant port data via the polarity correlator algorithm, which was presented in our earlier work [12].

## 2. Rotman Lens

### 2.1. Structure

**Figure 1:**
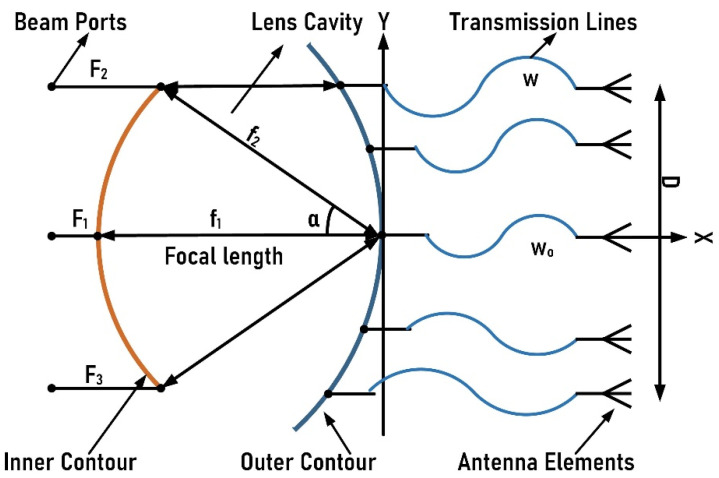
Basic schematic of a Rotman lens.

The Rotman lens has been widely used as a beamforming network. The schematic design of the Rotman lens is presented in Figure 1 [13]. The design consists of two contours forming a complete cavity of parallel plates. The inner contour normally contains M input ports, also known as the beam ports, while the outer contour contains N number of ports connected via routed or simple transmission lines to N number of array ports. When a signal is applied, it gets transmitted and received by all the ports. A progressive phase shift that is linear in nature is observed due to the disparities in the length of electrical lines among the output ports. This behavior makes a Rotman lens a true-time delay device with varying phase fronts at the array input via providing path delays within the lens cavity [14]. This path delay procedure is independent of any frequency [15], making the lens the best choice for various wideband applications that require wide-angle frequency scanning. In the lens design, conduction and absorption losses can be reduced by further adding dummy ports. From the schematic design of Figure 1, the main variables that contribute to the design are the tri-focal points denoted by F_1_, F_2_, and F_3_, and the main central focal length f_1_. On-axis focal point F_1_ and off-axis focal points F_2_ and F_3_ are located on the beam contour at angles of 0, +α, and −α, respectively [16].

### 2.2. Sensor Design

**Figure 2:**
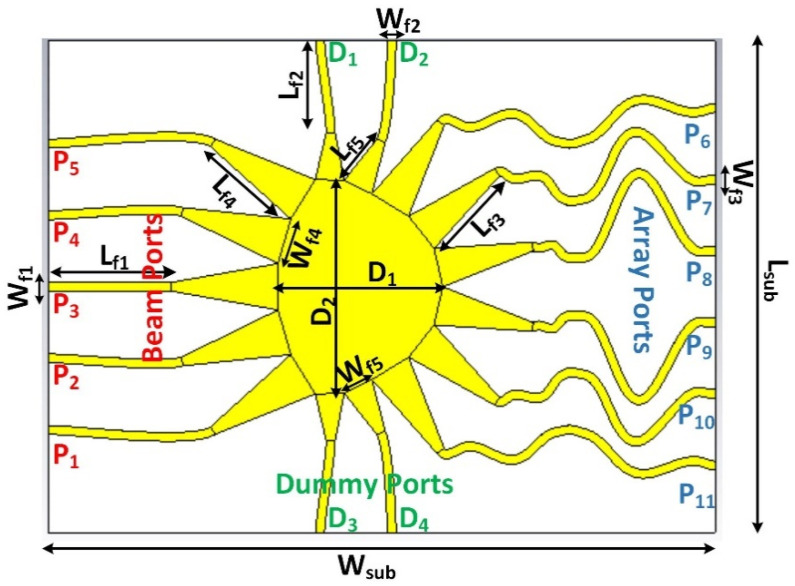
Geometric design of the proposed Rotman lens sensor.

The design of the proposed Rotman lens sensor is depicted in Figure 2. The design was realized on a Rogers RO4003C substrate with dielectric permittivity of 3.55, loss tangent of 0.0027, and a thickness of 0.8 mm. The design consists of a full ground plane on the rear side of the substrate, while the main routing transmission lines are formed on the upper side of the substrate. Multiple ports are distributed such that five beam ports are to the left side, six array ports are on the right side, and four dummy ports are designed and located on the top and bottom of the structure. The main central contour selected is in an elliptical shape for better beam propagation performance. The structure is designed to operate in the band of interest from 27 to 29 GHz with optimal dimensions provided in Table 1 focusing on the central frequency of 28 GHz. The design parameters were obtained via a commercial tool called Rotman Lens Designer (RLD) [17].

### 2.3. S-Parameters

**Figure 3:**
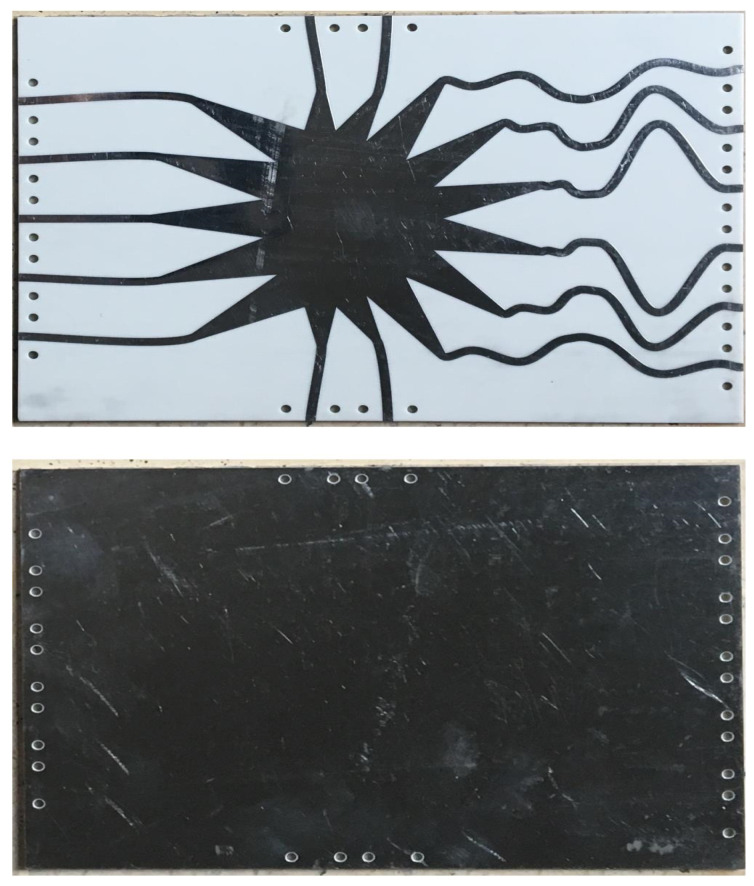
Realized prototype of the proposed Rotman lens sensor—front and back view.

**Figure 4:**
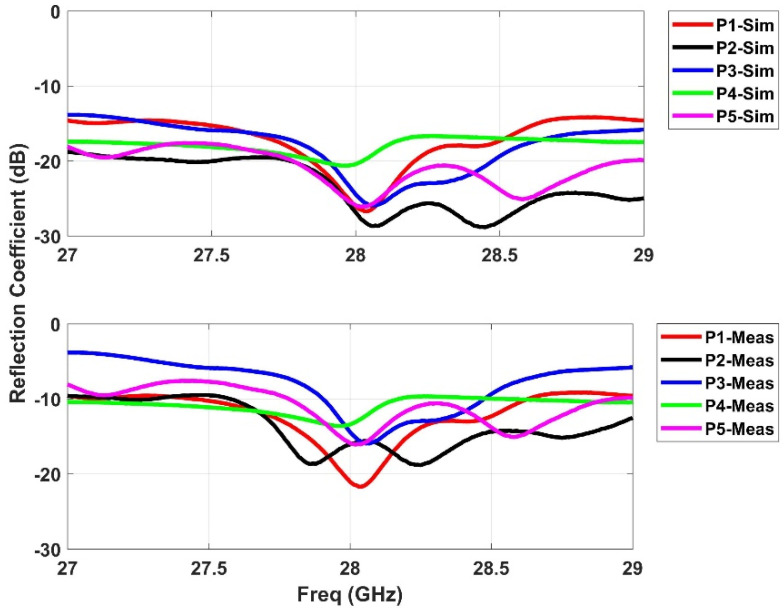
Simulated and measured s-parameters for the beam ports (P1–P5).

**Figure 5:**
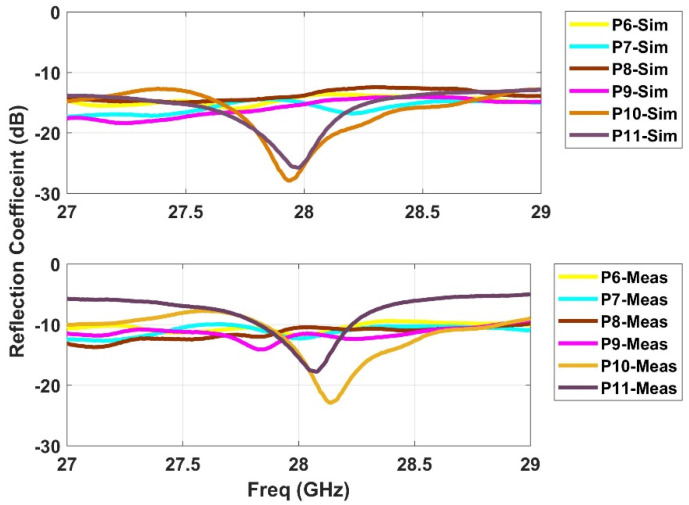
Simulated and measured s-parameters for the array ports (P6–P11).

**Figure 6:**
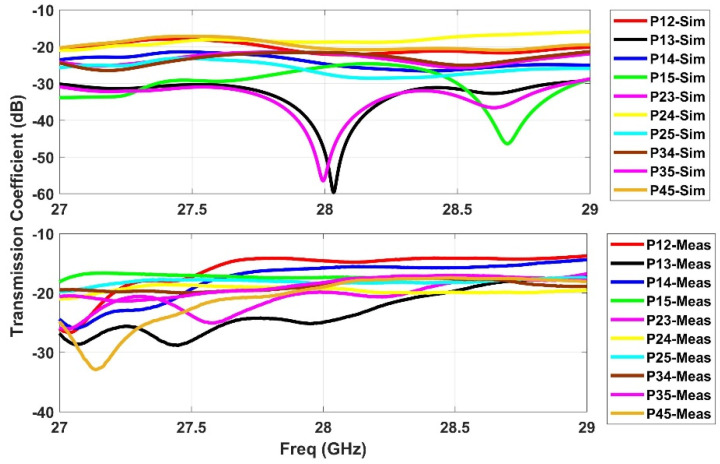
Simulated and measured s-parameters for the coupling among the beam ports (P1–P5).

**Figure 7:**
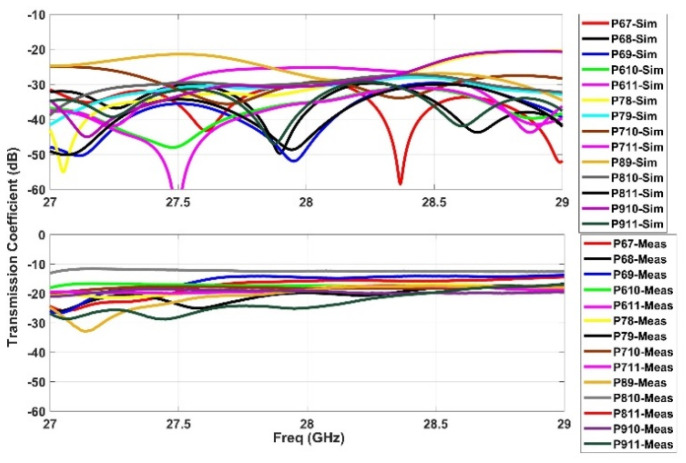
Simulated and measured s-parameters for the coupling among the array ports (P6–P11).

**Figure 8:**
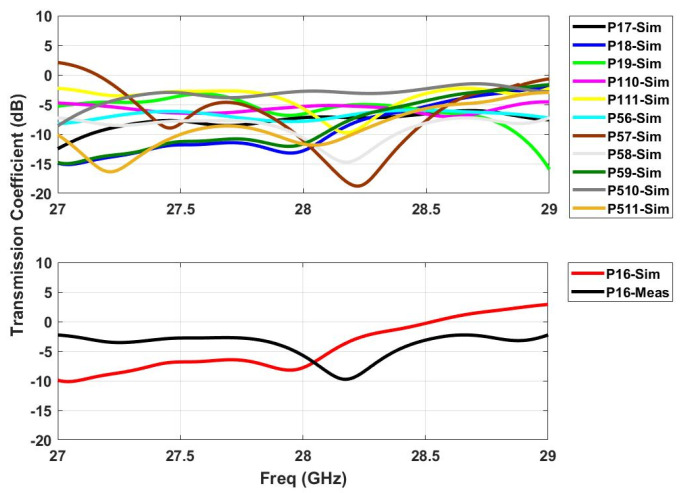
Simulated transmission coefficients (**top**) when ports 1 and 5 are active with measurement verification on a single port (P16) (**bottom**).

**Figure 9:**
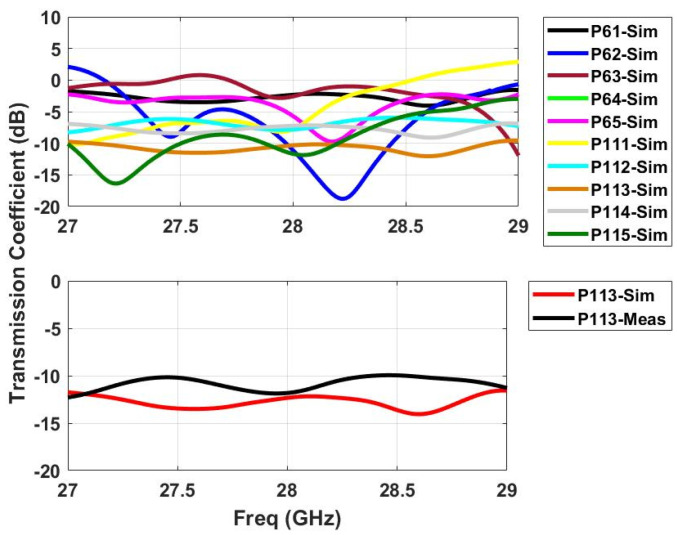
Simulated transmission coefficients (**top**) when ports 6 and 11 are active with measure-ment verification on a single port (P113) (**bottom**).

The realized prototype of the proposed Rotman lens sensor is presented in Figure 3. An LPKF Protomat E33 milling and cutting machine [18] was used to fabricate the structure. A high performance computation-based simulator, commercially known as Computer Simulation Technology (CST-MWS) [19], was used to rigorously compute the s-parameter performance of the proposed sensor. The main objective was to obtain a resonance in the band of interest from 27 to 29 GHz with central frequency being 28 GHz for mmW applications. Figure 4 depicts the beam port (P1–P5) performance of the simulator results with the measured response. It can be seen from the simulation results that the whole band of interest is being covered by the sensor. The measurements fairly confirm the simulation results, and interestingly, it can be observed that multiple responses of different behavior appear within the band of interest with acceptable matching at 28 GHz. Next, the s-parameters of the array port (P6–P11) were simulated, with the results presented in Figure 5. It is evident that the simulation results confirmed with measurements cover the entire band from 27 to 29 GHz. For the coupling analysis of the beam and array ports, Figure 6 and Figure 7 depict the simulated and measured results. It can be seen that the simulation results are well below the −20 dB reference line among the ports for both cases, while the measurement results are near −15 dB for the beam ports and array ports coupling with one signal near −12 dB for the array port coupling at P8–P10. The amplitude shifts in both the reflection and insertion loss could be due to poor soldering of the connectors with mutual coupling strength between the transmission lines. However, the main concern here is not the frequency shifts or dips above or below the reference lines of −10 or −20 dB. Similarly, the transmission coefficients of the proposed design are presented in Figure 8 and Figure 9. Figure 8 depicts the effect on all the ports in terms of its transmission coefficients when only ports 1 and 5 are activated. Figure 9 depicts the port response when ports 6 and 11 are active. It can be seen that the proposed Rotman lens sensor has transmission curves in the range of −4 to −15 dB. To verify the design, only single port measurements were taken, as shown in Figure 8 and Figure 9 for port P16 and port P113. The difference among the simulation and measurements was −4 dB for both ports, i.e., P16 and P113. The core intention was to analyze the complete mmW band of interest by exploiting the abundant data that were generated and collected from all the ports of the proposed Rotman lens sensor. This data can then be utilized via signal processing algorithms for better characterization accuracy.

### 2.4. Surface Current Distributions

**Figure 10:**
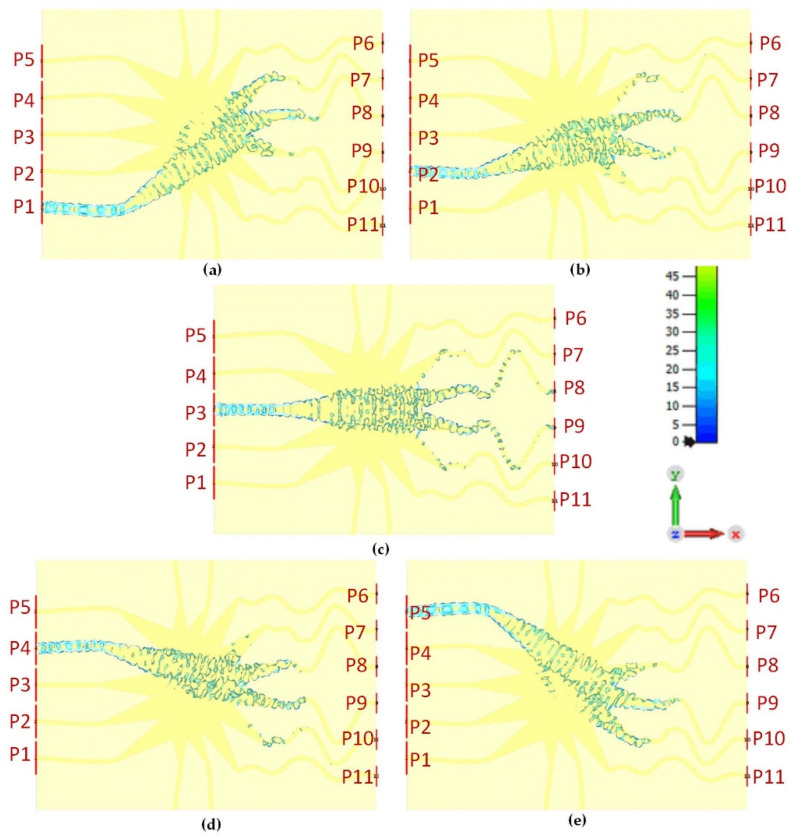
Surface current distributions of the beam port (P1–P5), from below to above, for the proposed Rotman lens sensor; (**a**) P1 is active, (**b**) P2 is active, (**c**) P3 is active, (**d**) P4 is active, (**e**) P5 is active.

**Figure 11:**
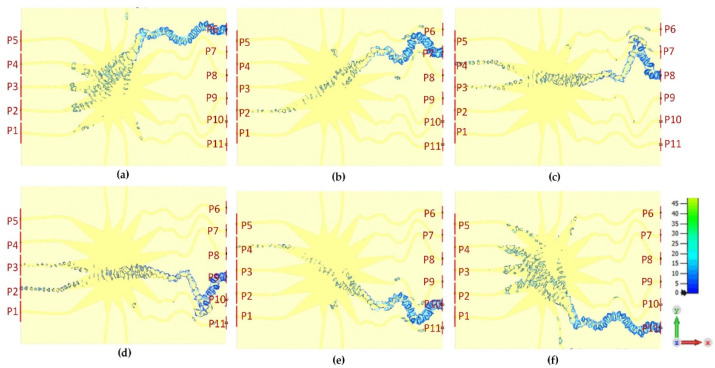
Surface current distributions of the array port (P6–P11), from below to above, for the proposed Rotman lens sensor; (**a**) P6 is active, (**b**) P7 is active, (**c**) P8 is active, (**d**) P9 is active, (**e**) P10 is active, (**f**) P11 is active.

Figure 10a–e represents the surface current distributions of the proposed Rotman lens sensor for all the five beam ports (P1–P5). It can be seen that when each port is excited individually, the wave gets propagated from the port, follows the main elliptical contour, and reaches the corresponding array ports on the right-hand side of the substrate. For each excitation, wave energy gets transmitted and absorbed into the port in close proximity to the array ports. Similarly, Figure 11a–f depicts the surface current distributions of the array ports (P6–P11). Each array port gets excited individually, with EM energy travelling from the elliptical contour to the beam ports. A few absorptions are seen near the coupling array ports with some energy going into the dummy ports. The overall surface current distributions confirm that the proposed Rotman lens sensor properly propagates the EM wave between all the ports.

## 3. Sample Load for Testing the Sensor

### 3.1. Material Preparation

**Figure 12:**
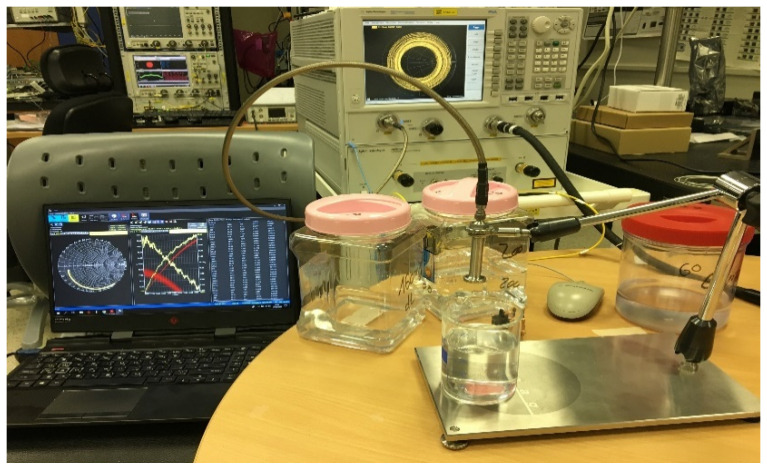
Solutions being tested for their material characterization via SPEAG DAK tool.

Various in-lab solutions were prepared in order to verify that the proposed Rotman lens can work as a sensor. Aqueous glucose solution was utilized as a loading element due to its popularity in the literature [20] and ease of preparation and procurement. The concentrations selected for the experiment were of 60, 100, and 200 mg/dL as depicted in Figure 12. Dextrose anhydrous powder was used in the preparation of the aqueous solution as it is easily available and widely used in the food industry [21]. Moreover, it is an optimal powder in testing a liquid-based solution compared to fructose. Homogeneity of the proposed solutions was ensured by keeping a defined room temperature and a constant spinning speed [22]. The solutions were characterized by utilizing a SPEAG [23] dielectric testing kit (DAK).

### 3.2. Sensor Validation

**Figure 13:**
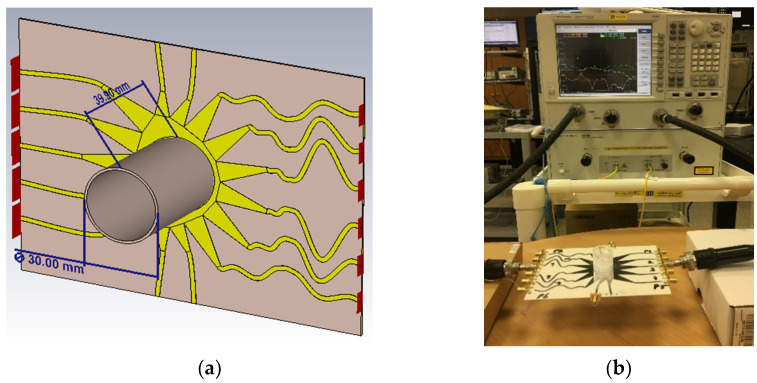
Testing the sensor: (**a**) Placement of the beaker onto the Rotman lens with the cylinder’s geometrical dimensions; (**b**) Characterization of the proposed sensor.

**Figure 14:**
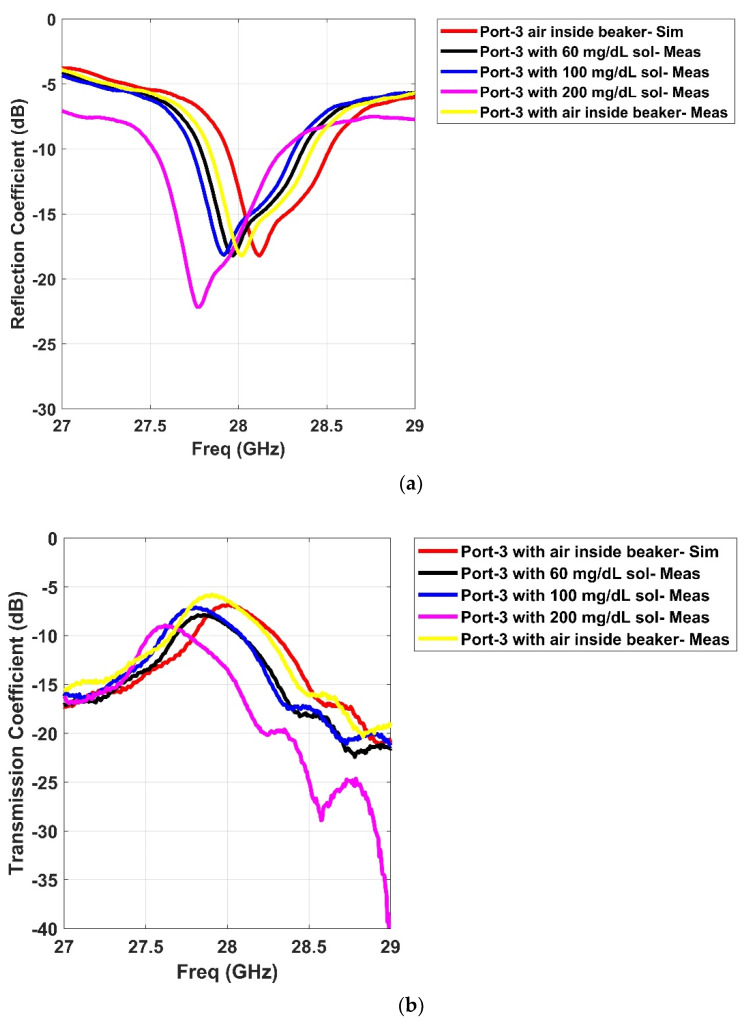

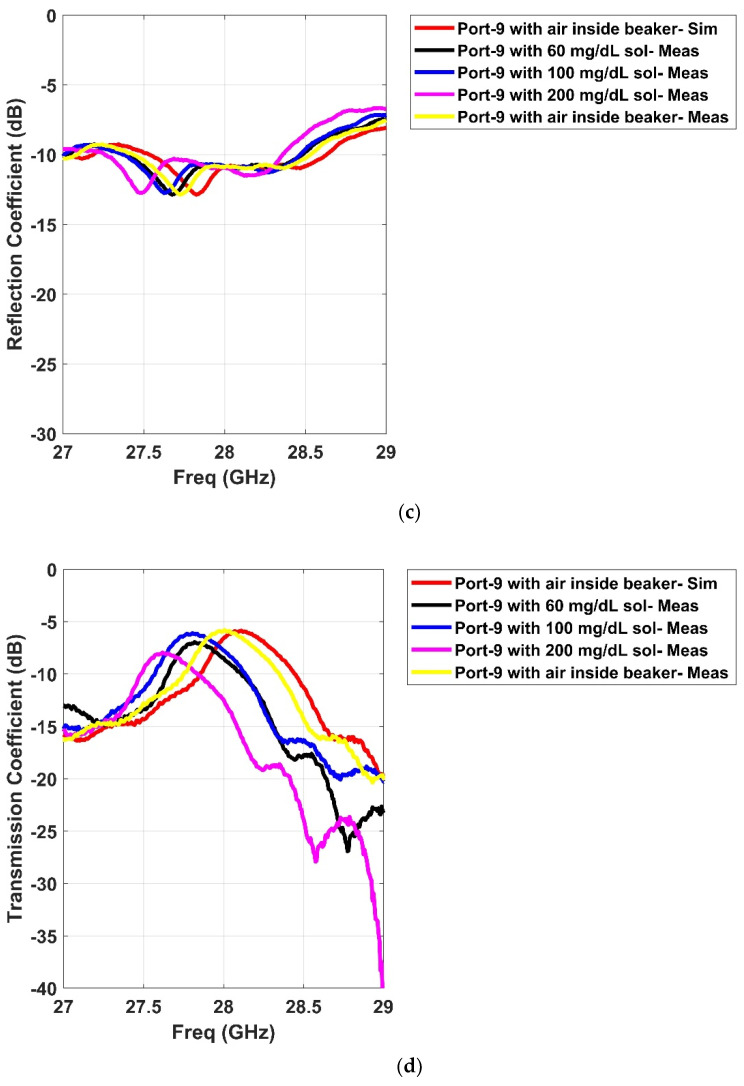
Comparison of simulated and measured responses of the proposed Rotman lens sensor after loading with prepared in-lab aqueous solutions: (**a**) Port 3 reflection coefficient; (**b**) Port 3 transmission coefficient between P39; (**c**) Port 9 reflection coefficient; (**d**) Port 9 transmission coeff-cient between P93.

The proposed sensor was tested with the prepared in-lab solutions. The solutions were injected with a medical syringe inside the cylindrical beaker at appropriate height of 0.3 cm and room temperature of 25 °C. Figure 13a shows the beaker. Its geometric dimensions were an upper diameter of 30 mm and a height of 39 mm. It was placed onto the Rotman lens, and Figure 13b depicts the characterization of the realized prototype. The measurements were taken via a vector network analyzer (VNA) [24]. The placement of the liquids onto the sensor generated different signal behavior over the entire band of interest due to multiple ports as presented earlier for the s-parameter results. In order for the Rotman lens to work as a sensor, it needs to introduce variations in port responses including the presence of some nulls in the band of interest. In our analysis, we measured the different resonance behaviors of only two ports (P3–P9) for each aqueous concentration including air and solutions (60, 100, and 200 mg/dL). Figure 14 presents the various resonance behaviors (reflection and transmission coefficient) observed in comparison between simulated and measured results for the proposed sensor. It can be seen from Figure 14a that when the beaker was placed with air inside (no solution), the simulated response, shown by the red curve, covered the entire band of interest, i.e., from 27 to 29 GHz. The measured response of the same red curve, shown by the dotted yellow line, also covered the entire band. Similarly, when the solutions were added to the cylindrical beaker at 60 (black dotted curve), 100 (blue dotted curve), and 200 mg/dL (pink dotted curve), different resonance behaviors were observed. Specifically, the nulls of the aqueous solutions were analyzed where it was seen that the measured curves moved towards the lower end of the frequency band with an increase in the aqueous concentrations. This behavior was also observed in the transmission coefficient response of the sensor when port 3 was active, as shown in Figure 14b. When there was no solution and just air inside the beaker, the transmission coefficients were near the −5 dB resonance. With the addition of solutions to the beaker, the sensor’s response became weak as the resonance level decreased from −5 to around −10 dB. This decrease may be due to the coupling among the multiple ports, but still the measurements were in acceptable ranges of transmission coefficients for mmW bands. Similarly, as seen in Figure 14c,d, analysis based on the measurement for port 9 with prepared in-lab solutions reveal that the beaker with no solution (air) covered the entire band from 27 to 29 GHz (red simulated curve), while the measured (yellow dotted curve) also covered the same band. For the solutions at 60 (black dotted curve), 100 (blue dotted curve), and 200 mg/dL (pink dotted curve), again, null shifting towards the lower end of the frequency bands was observed. Table 2 presents the null values for each of the measured ports as per loading of the aqueous solutions. In conclusion, for both the ports, a shift towards the lower end of the spectrum was observed when higher concentrations were added to the beaker. This proves that the sensor based on the Rotman lens design performs in somewhat similar manner to other available RF sensors in the literature when loaded with different materials [25]. The added advantage with this design is that abundant data could be gathered from the multiple ports as per lens geometry and can be utilized by various signal processing and artificial intelligence algorithms in providing more insight into liquid characterization.

## 4. Port Data Analysis

The polarity correlator previously introduced in [12] can be used to analyze the responses of the Rotman lens when the lens works as a sensor. As discussed in [12], the polarity correlator exploits the variations present in the whole response of the sensor in order to differentiate between different concentrations by measuring the similarity of a sensor’s response to an unknown concentration with pre-stored sensor responses to known concentrations. To provide comprehensive analysis for all possible Rotman lens responses, this section presents results produced only by simulation. This is because the Rotman lens has five beam ports and six array ports; hence, the number of possible lens responses is 66. The numbering and symbols denoting these responses at particular concentrations are listed in Table 3. For example, P5–P1 (22) means that the response between ports 5 and 1 is given the number 22. The polarity correlator works on the sign of filtered responses, as described below.

Let $s_{i}^{m}\left(k\Delta f\right)$ denote the *i*th response (*i* = 1,2, …, 66) corresponding to a solution of concentration *m*. The symbol *k* denotes the *k*th response sample, and Δf is the sampling interval in the frequency domain. The pulse fidelity factor is used here to differentiate between pre-processed output responses of two different concentrations *m* and *n*. The pre-processed response $r_{i}^{m}\left(k\Delta f\right)\;$is obtained as follows:

(1) $$r_{i}^{m}\left(k\Delta f\right)\;=\;\mathrm{sign}\left(s_{i}^{m}\left(k\Delta f\right)-s_{i}^{m}\left(\left(k-1\right)\Delta f\right)\right)$$

where sign(*x*) = 1 if *x* > 0 and −1 if *x* < 0. This pre-processing step improves the discrimination between the different concentrations, as previously demonstrated in [12]. The pulse fidelity factor $p_{i}^{mn}$ is given by:

(2) $$p_{i}^{mn}=\begin{array}{c} \max\\ k_{0} \end{array}\frac{\sum_{k}r_{i}^{m}\left(k\Delta f\right)r_{i}^{n}\left(\left(k-k_{0}\right)\Delta f\right)}{\sqrt{\sum_{k}{\left|r_{i}^{m}\left(k\Delta f\right)\right|}^{2}\sum_{k}{\left|r_{i}^{n}\left(k\Delta f\right)\right|}^{2}}}.$$

The polarity correlator implements both operations of Equations (1) and (2). In our development, the pulse fidelity factor is used to measure the similarity between two pre-processed responses produced by the Rotman lens at three different glucose concentrations (*m*,*n* = 1, 2, 3). These concentrations are produced by pure water (*m* = *n* = 1), and water with glucose concentrations of 60 (*m* = *n* = 2) and 100 mg/dL (*m* = *n* = 3). Note that for better discrimination, the fidelity factor $p_{i}^{mn}$ between the responses of two different concentrations (*m* ≠ *n*) should be as small a value as possible (i.e., close to zero). For *m* = *n*, the fidelity factor is identically 1.

**Figure 15:**
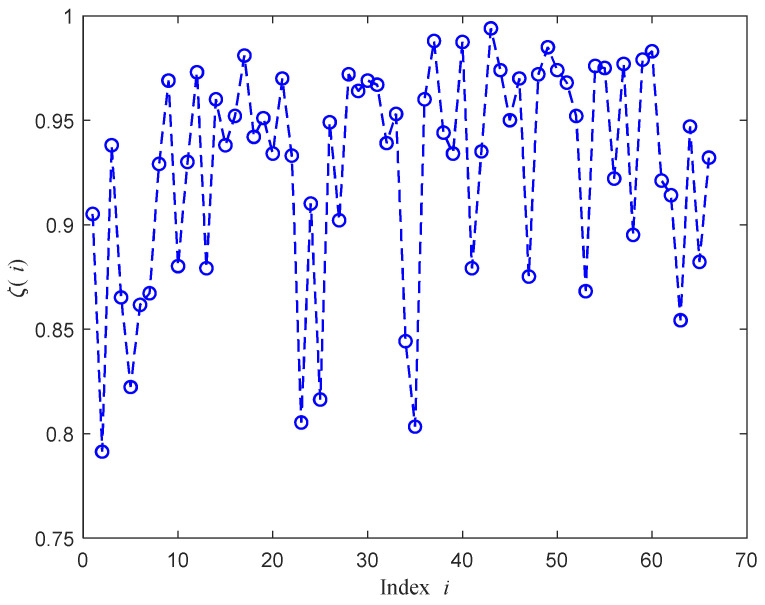
The maximum of fidelity factor versus the response index *i*.

Figure 15 shows the maximum value of fidelity factor, denoted by ζ(i), of the *i*th response computed for *m*,*n* = 1, 2, 3 (*m* ≠ *n*). That is,

(3) $$\zeta \left(i\right)\;=\;\max\left\{p_{i}^{12},\;p_{i}^{13},\;p_{i}^{23}\right\},i=1,2,3,\;\ldots ,66.$$

**Figure 16:**
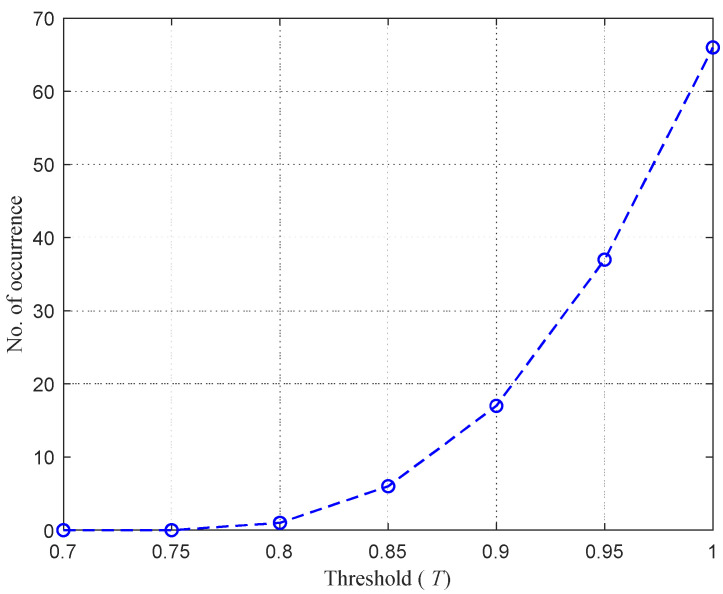
Number of responses that have maximum fidelity factor less than or equal to the given threshold *T*.

**Figure 17:**
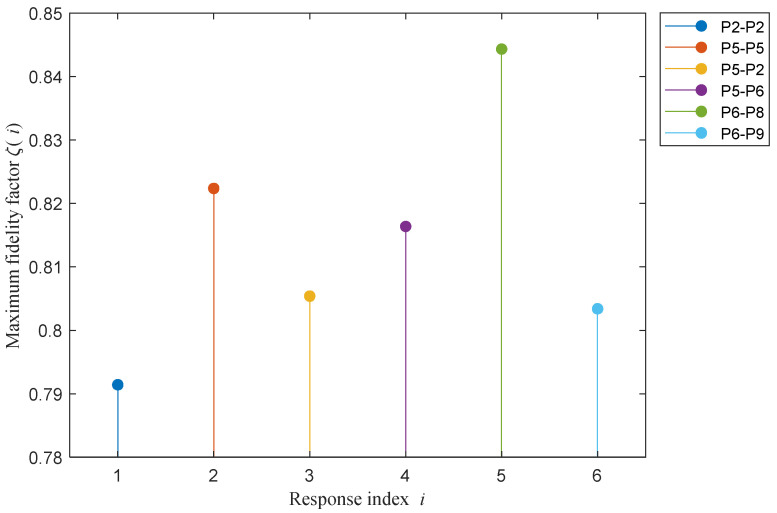
Responses that have maximum fidelity factor less than or equal to 0.85.

It is observed from the figure that the fidelity factor varies among responses of different ports. Ports that have lower values of ζ(i) are highly preferred to differentiate between given concentrations. Figure 16 shows the number of responses that have values of ζ(i) less than or equal to a given threshold, *T*. Table 4 gives the number of responses, the ζ(i) of which represents values less than or equal to *T* = 0.75, 0.8, 0.85, 0.9, 0.95, and 1. For example, six different responses have maximum fidelity factor with values less than or equal to 0.85. These responses corresponding to the index *i* = 2, 5, 23, 25, 34, and 35 are obtained from ports P2-P2, P5-P5, P5-P2, P5-P6, P6-P8, and P6-P9, with Figure 17 showing their ζ(i) versus the response index *i*. Therefore, we can consider the determination of a concentration value as a classification problem, along the lines of the approach reported in [12], but with additional steps exploiting data of more than one port of the Rotman lens. Specifically, the following steps were performed.

▪ Pre-measured responses of *N* solutions with different concentration values were stored in a library. Note that each solution has six responses corresponding to *i* = 2, 5, 23, 25, 34, and 35.
▪ The fidelity factor between the response of a solution of unknown concentration and the 6*N* responses of *N* pre-stored solutions were computed.

The concentration of pre-stored response, the fidelity factor of which with the response to unknown solution is the maximum, is considered the unknown concentration of the solution under measurement. Note that the use of the Rotman lens increased the reliability of concentration determination as the number of responses with which the unknown response was cross-correlated increased sixfold. For more accurate results, more responses of the Rotman lens can be seen in Table 4. For *T* = 0.9, there are 17 responses, which can be exploited in determining the unknown concentration.

## 5. Conclusions

In this effort, and for the first time, we investigated the performance of a Rotman lens as a sensor. The proposed sensor operates in the mmW band with a center frequency of 28 GHz. The Rotman lens sensor consists of five beam ports, six array ports, and four dummy ports. Each port was analyzed in terms of its s-parameters and mutual coupling. A cylindrical beaker can be placed as a loading element in the central elliptical position of the Rotman lens sensor. Three in-lab aqueous solutions were prepared. Simulated and measured responses of the Rotman lens ports were analyzed and compared in terms of s-parameters. It was demonstrated that the use of the Rotman lens increases the reliability of determining the unknown concentration of a given solution by exploiting data of more than one port and through the use of a polarity correlator.

## Figures and Tables

**Table 1:** Optimized dimensions of the proposed Rotman lens sensor operating in the mmW band.

Parameter	Value (mm)	Parameter	Value (mm)
L_sub_	140	W_sub_	103
L_f1_	35	W_f1_	1.85
L_f2_	20.54	W_f2_	1.85
L_f3_	20.66	W_f3_	1.85
L_f4_	22.67	W_f4_	9.73
L_f5_	10.16	W_f5_	6.48
D_1_	34.9	D_2_	45.22

**Table 2:** Shifting null values for ports 3 and 9 measurements based on loading Rotman lens sensor with aqueous solution.

Aqueous Solutions	Nulls @ Port 3	Aqueous Solutions	Nulls @ Port 9
Air	28.01 GHz	Air	27.7 GHz
60 mg/dL	27.96 GHz	60 mg/dL	27.67 GHz
100 mg/dL	27.91 GHz	100 mg/dL	27.63 GHz
200 mg/dL	27.77 GHz	200 mg/dL	27.47 GHz

**Table 3:** Numbering and symbols denoting responses of the Rotman lens.

P1-P1 (1), P2-P2 (2), P3-P3 (3), P4-P4 (4), P5-P5 (5), P6-P6 (6), P7-P7 (7), P8-P8 (8), P9-P9 (9), P10-P10 (10), P11-P11 (11), P2-P1 (12), P3-P1 (13), P3-P2 (14), P4-P1 (15), P4-P2 (16), P4-P3 (17), P4-P5 (18), P4-P6 (19), P4-P7 (20), P4-P10 (21), P5-P1 (22), P5-P2 (23), P5-P3 (24), P5-P6 (25), P5-P7 (26), P5-P8 (27), P5-P9 (28), P5-P10 (29), P5-P11 (30), P6-P1 (31), P6-P2 (32), P6-P3 (33), P6-P8 (34), P6-P9 (35), P6-P10 (36), P6-P11 (37), P7-P1 (38), P7-P2 (39), P7-P3 (40), P7-P6 (41), P7-P8 (42), P8-P1 (43), P8-P2 (44), P8-P3 (45), P8-P4 (46), P8-P10 (47), P8-P11 (48), P9-P1 (49), P9-P2 (50), P9-P3 (51), P9-P4 (52), P9-P7 (53), P9-P8 (54), P9-P10 (55), P9-P11 (56), P10-P1 (57), P10-P2 (58), P10-P3 (59), P10-P7 (60), P10-P11 (61), P11-P1 (62), P11-P2 (63), P11-P3 (64), P11-P4 (65), P11-P7 (66)

**Table 4:** Number of responses with maximum fidelity factor less than or equal to the threshold *T*.

Threshold (*T*)	0.75	0.8	0.85	0.9					0.95	1
Number of responses			0	1	6	17	37	66		

## Data Availability

Data available on request from the authors.
